# Etanercept decreases HMGB1 expression in dorsal root ganglion neuron cells in a rat chronic constriction injury model

**DOI:** 10.3892/etm.2012.810

**Published:** 2012-11-13

**Authors:** RUI-KE WANG, QIN-QIN ZHANG, YUN-DAN PAN, QU-LIAN GUO

**Affiliations:** Department of Anesthesiology, Xiangya Hospital, Central South University, Changsha, Hunan 410008, P.R. China

**Keywords:** etanercept, high mobility group box 1, tumor necrosis factor-α, chronic constriction injury, dorsal root ganglion, mitogen-activated protein kinase

## Abstract

In the present study, we examined the effect of etanercept on high mobility group box 1 (HMGB1) expression in dorsal root ganglion (DRG) neuron cells in a rat model of chronic constriction injury (CCI) of the sciatic nerve, with the aim of exploring the molecular mechanism underlying the therapeutic effect of etanercept on sciatica-related nociception and the potential interaction between tumor necrosis factor-α (TNF-α) and HMGB1 in DRG neuron cells. A rat CCI model was employed and the animals were randomly assigned to seven groups (n=20/group): untreated, sham only, sham/saline, sham/etanercept, CCI only, CCI/saline and CCI/etanercept. Our results revealed that compared with the sham/saline and sham/etanercept groups, thermal hyperalgesia and mechanical hyperalgesia, as well as HMGB1 expression at both the mRNA and protein levels in the DRG neuron cells, were induced by CCI, and were significantly inhibited by etanercept. Although etanercept showed no significant effect on the sham group, it significantly reduced the phosphorylated p38 mitogen-activated protein kinase (MAPK) levels induced by CCI in the DRG neuron cells. In conclusion, we demonstrated that etanercept significantly decreased the HMGB1 expression induced by CCI in the DRG neuron cells. This study not only explored the molecular mechanisms underlying the therapeutic effect of etanercept on sciatica-related nociception, but also provided indirect evidence for an interaction between TNF-α and HMGB1 in DRG neuron cells.

## Introduction

Previous experimental evidence suggests that nerve root inflammation, with or without concomitant nerve compression, is an important contributing factor to sciatica or radicular pain (1). Human herniated discs have been shown to express a number of pro-inflammatory mediators, including tumor necrosis factor-α (TNF-α), interleukin-1 (IL-1), IL-6 and IL-8 (1). TNF-α and its receptor are reportedly upregulated in dorsal root ganglion (DRG) neurons following lumbar injury in rats (2,3). The application of recombinant TNF-α to the lumbar nerve roots of rodents induces mechanical allodynia and hyperalgesia (1). Etanercept, a recombinant TNF receptor (p75)-Fc fusion protein, competitively inhibits TNF-α (4). Sommer *et al*(4) reported that etanercept reduced pain and hyperalgesia in a rat model of painful neuropathy induced by the chronic constriction injury (CCI) of the sciatic nerve.

High mobility group box 1 (HMGB1) is a part of the nucleic acid-sensing system and binds to immunogenic nucleotides in order to activate innate immune responses during microbial infection and tissue damage (5). Biologically active HMGB1 is expressed on the plasma membrane or is secreted into the extracellular milieu where it acts as a cytokine and interacts with the receptor for advanced glycation end products (RAGE) and toll-like receptor (TLR)-2, TLR4 and TLR9 (6,7). It has been reported that the induction of HMGB1 in DRGs contributes to pain hypersensitivity following peripheral nerve injury (8) and an anti-HMGB1 neutralization antibody improves pain-related behavior induced by the application of autologous nucleus pulposus onto nerve roots in rats (9). It has been suggested that HMGB1 plays an important role in the pathophysiology of CCI and sciatica-related nociception.

In the present study, we for the first time examined the effect of etanercept on HMGB1 expression in DRG neuron cells in a rat CCI model, with the aim of exploring the molecular mechanism underlying the therapeutic effect of etanercept on sciatica-related nociception and the potential interaction between TNF-α and HMGB1 in DRG neuron cells.

## Materials and methods

### Animals

Male inbred Sprague-Dawley rats (weight 250–300 g) were purchased from Central South University and were housed at the Xiangya Hospital BioResources Centre. Animals were placed in a quiet, temperature (22±2°C) and humidity (60±6%) controlled room with a 12:12 h light-dark cycle (light beginning at 8 a.m.) and all tests were performed during the light phase of the cycle. Pharmaceutical-grade etanercept was purchased from Amgen (Thousand Oaks, CA, USA). Anti-HMGB1 (sc-12523) antibody was purchased from Santa Cruz Biotechnology, Inc. (Santa Cruz, CA, USA). Anti-p38 mitogen-activated protein kinase (p38 MAPK; #8690), anti-phospho-p38-MAPK (Thr180/Tyr182; #9211) and anti-glyceraldehyde-3-phosphate dehydrogenase (GAPDH; #2118) antibodies were purchased from Cell Signaling Technology (Beverly, MA, USA). All secondary antibodies were from Jackson ImmunoResearch Laboratories (West Grove, PA, USA). All chemicals of reagent grade were purchased from Sigma (St. Louis, MO, USA).

### Establishment of rat CCI model and treatment groups

CCI was induced according to the method of Bennett and Xie (10). Briefly, each animal was anesthetized by an intraperitoneal injection of sodium pentobarbital at a dose of 60 mg/kg. The common sciatic nerve was exposed and freed from adherent tissue at the mid-thigh by separating the biceps femoris muscles by blunt dissection. Four loose ligatures were placed 1 mm apart using chromic gut suture (4-0 absorbable suture; Jorgensen Laboratories, Inc., Loveland, CO, USA). The animals were randomly assigned to seven groups (n=20/group): untreated, sham only (animals subjected to sham surgery only), sham/saline (animals subjected to sham surgery plus intrathecal injection of 20 *μ*l saline every two days from two days before surgery), sham/etanercept [animals subjected to sham surgery plus intrathecal injection of 20 *μ*l (100 *μ*g) etanercept every two days from two days before surgery], CCI only (animals subjected to CCI surgery only), CCI/saline (animals subjected to CCI surgery plus intrathecal injection of 20 *μ*l saline every two days from two days before surgery) and CCI/etanercept group [animals subjected to CCI surgery plus intrathecal injection of 20 *μ*l (100 *μ*g) etanercept every two days from two days before surgery]. This study was conducted in accordance with our institutional guidelines on the use of live animals for research and the experimental protocol was approved by the Laboratory Animal Users Committee at Xiangya Hospital, Central South University.

### Behavior examination

Thermal hyperalgesia was measured according to the Hargreaves test (1) using a plantar analgesia instrument (Stoelting, Wood Dale, IL, USA) every day from one day before surgery to 13 days after surgery. The radiant infrared heat source stimulus intensity was set to IR50 and the cut-off time was set at 15 sec. The rats were placed on a glass platform and allowed to habituate to the testing chambers for a minimum of 15 min prior to each testing session. The thermal stimulus was applied to the plantar surface of the paw. Thermal thresholds were defined as the latency in seconds at the first pain behavior, which includes paw withdrawal, flinching, biting and/or licking of the stimulated paw. The readings for all animals were averaged and the mean and standard error of the mean were determined for each treatment group. Mechanical allodynia was measured using von Frey monofilaments (Stoelting) with varying stiffness (2.0–15.0 g) every day from one day before surgery to 13 days after surgery. The rats were placed on a perforated metallic platform and allowed to habituate to their surroundings for a minimum of 15 min before testing. The paw withdrawal threshold response was determined by a sequential increasing and/or decreasing of the stimulus strength.

### DRG neuron cell isolation and real-time quantitative reverse transcription-polymerase chain reaction (RT-PCR)

On days 3, 7 and 13 after surgery, four randomly selected rats were sacrificed at each time point and DRG neuron cells were isolated from the enlarged part of the lumbar spinal cord as previously described (11). Cells were used for experiments 24–48 h after isolation. DRG neurons used for mRNA extraction were stored at −80°C immediately after isolation. RNA samples were prepared using TRIzol reagent followed by purification with Turbo DNA-free system (Ambion, Austin, TX, USA). The cDNAs were synthesized using SuperScript II reverse transcriptase (Invitrogen, Carlsbad, CA, USA). Real-time quantitative PCR was performed on the LightCycler thermal cycler system (Roche Diagnostics, Indianapolis, IN, USA) using a SYBR-Green I kit (Roche Diagnostics) as described by the manufacturer. Each result was normalized against that of the housekeeping gene GAPDH in the same sample. The primers used were as follows: for rat HMGB1, 5′-GTACGGTACCAAGTGCATTT TGGAGGAATT-3′ (forward) and 5′-GTACAAGCTTGTACT GCAATGGCTGTGAGA-3′ (reverse) and for rat GAPDH, 5′-AAGCCCATCACCATCTTCCA-3′ (forward) and 5′-CCTGCTTCACCACCTTCTTG -3′ (reverse). Each experiment was repeated twice in triplicate.

### Western blot analysis

Immunoblotting was performed as described previously with respective antibodies (12). Briefly, DRG neuron cells were lysed in 0.1% Nonidet P-40 lysis buffer [(0.1% Nonidet P-40, 50 mM Tris-HCl (pH 7.4), 150 mM NaCl and 1 mM ethylenediamine tetraacetic acid (EDTA)]. Equal amounts of lysates were loaded onto 10% sodium dodecyl sulphate (SDS)-polyacrylamide gels and the proteins were blotted onto a polyvinylidene difluoride microporous membrane (Millipore, Billerica, MA, USA). The membranes were incubated for 1 h with a 1/1,000 dilution of anti-HMGB1, anti-p38 MAPK, anti-phospho-p38 MAPK (Thr180/Tyr182) or anti-GAPDH antibodies and then washed and revealed using secondary antibodies with a horseradish peroxidase conjugate (1/5,000, 1 h). Peroxidase was revealed with an ECL kit (GE Healthcare Life Sciences, Piscataway, NJ, USA). The proteins were quantified before being loaded onto the gel and equal loading of extracts was verified by Ponceau coloration.

### Statistical analysis

Statistical analyses were performed with SPSS (SPSS Inc., Chicago, IL, USA) for Windows 10.0. Data values were expressed as the mean ± standard deviation. Comparisons of means among multiple groups were performed with one-way analysis of variance (ANOVA) followed by post hoc pairwise comparisons using the least significant difference method. P<0.05 was considered to indicate a statistically significant difference.

## Results

**Figure 1.**
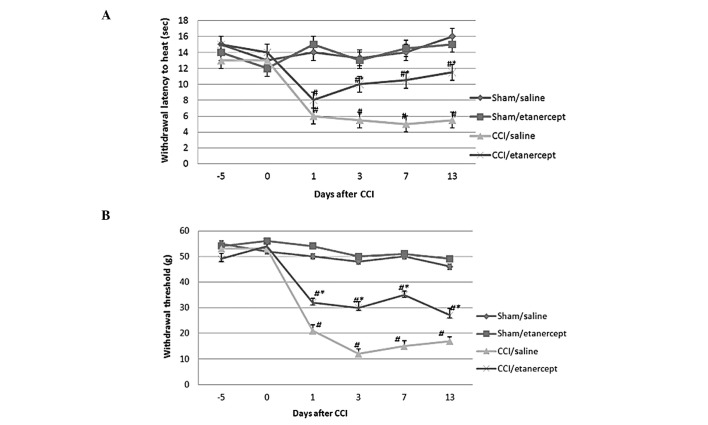
Evaluation of thermal hyperalgesia (A) and mechanical allodynia (B) in rats on days 0, 1, 3, 7 and 13 of chronic constriction injury (CCI) of the sciatic nerve. In the sham/saline and sham/etanercept groups, the rats were subjected to sham surgery plus intrathecal injection of 20 *μ*l saline or 20 *μ*l (100 *μ*g) etanercept, respectively, every two days from two days before surgery; in the CCI/saline and CCI/etanercept groups, the rats were subjected to CCI surgery plus intrathecal injection of 20 *μ*l saline or 20 *μ*l (100 *μ*g) etanercept, respectively, every two days from two days before surgery ^#^P<0.05 vs. the sham/saline group; ^*^P<0.05 vs. the CCI/saline group (n=7).

As shown in Fig. 1, compared with the sham/saline and sham/etanercept groups, thermal and mechanical hyperalgesia were induced by CCI on all testing days. Although etanercept showed no significant effect on paw withdrawal latency and threshold in the sham group, it significantly inhibited the thermal and mechanical hyperalgesia induced by CCI. The untreated and sham only groups showed no significant difference from the sham/saline and the sham/etanercept groups. The CCI only group showed no significant difference from the CCI/saline group (data not shown).

**Figure 2.**
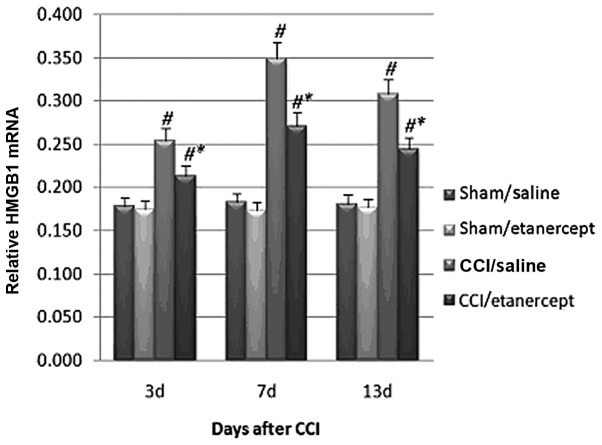
High mobility group box 1 (HMGB1) mRNA levels in rat dorsal root ganglion (DRG) neuron cells following chronic constriction injury (CCI) of the sciatic nerve. The relative HMGB1 mRNA level in the rat DRG neuron cells was determined by real-time reverse transcription-polymerase chain reaction (RT-PCR) on day 1, 3 and 13 of CCI. ^#^P<0.01 vs. the sham/saline group; ^*^P<0.01 vs. the CCI/saline group (n=4).

Real-time RT-PCR revealed that compared with those in the sham/saline and sham/etanercept groups, the HMGB1 mRNA levels in the DRG neuron cells were significantly increased by CCI on all testing days (Fig. 2). Treatment with etanercept showed no significant effect on the sham group. By contrast, it significantly reduced CCI-induced HMGB1 mRNA level (Fig. 2).

**Figure 3.**
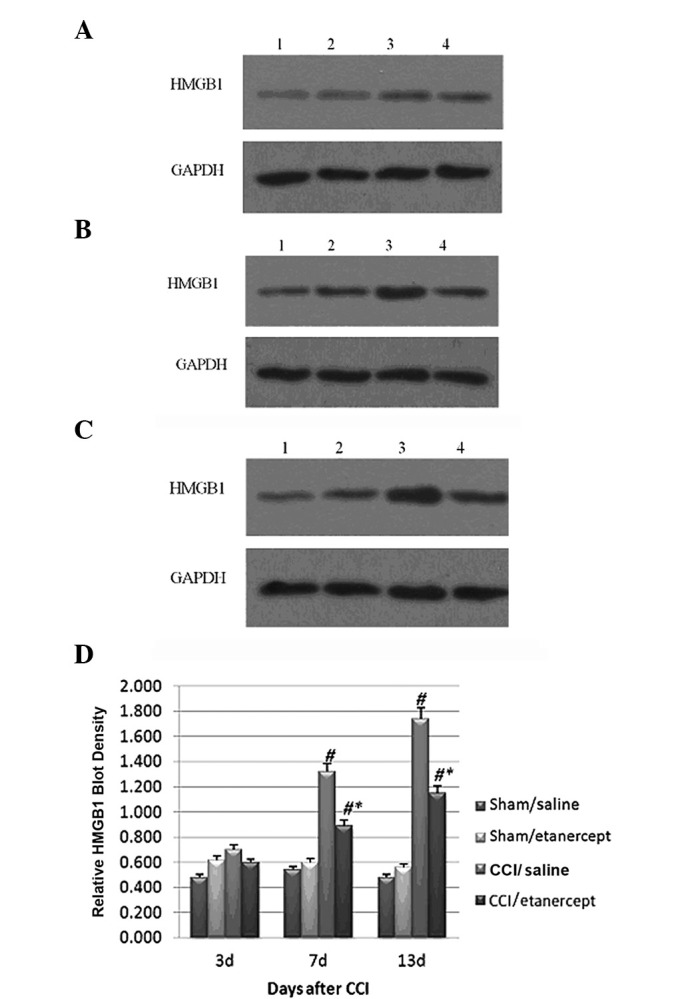
High mobility group box 1 (HMGB1) protein expression in rat dorsal root ganglion (DRG) neuron cells following chronic constriction injury (CCI) of the sciatic nerve. HMGB1 protein expression in the rat DRG neuron cells was determined by western blot analysis on days (A) 1, (B) 3 and (C) 13 of CCI. Glyceraldehyde-3-phosphate dehydrogenase (GAPDH) blotting was used as a loading control. Protein blots were measured by densitometry. (D) The density of each HMGB1 blot was normalized against that of GAPDH to obtain a relative HMGB1 blot density. Lane 1, sham/saline; lane 2, sham/etanercept; lane 3, CCI/saline; lane 4, CCI/etanercept. ^#^P<0.01 vs. the sham/saline group; ^*^P<0.01 vs. the CCI/saline group (n=4).

**Figure 4.**
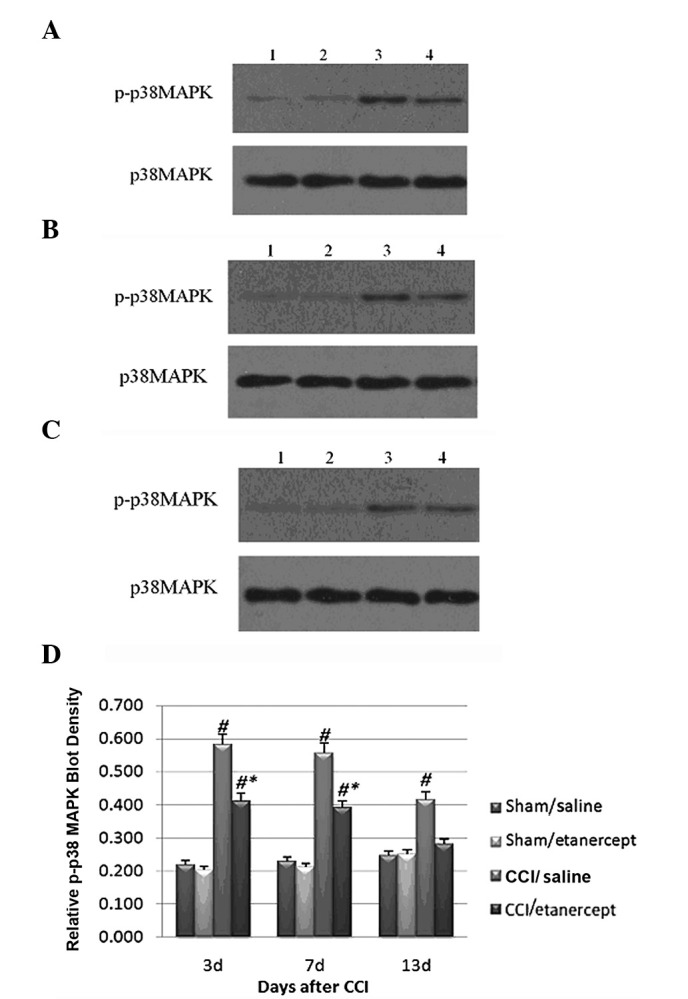
Phosphorylated p38 mitogen-activated protein kinase (p-p38 MAPK) level in rat dorsal root ganglion (DRG) neuron cells following chronic constriction injury (CCI) of the sciatic nerve. Total p38 MAPK and p-p38 MAPK levels in the rat DRG neuron cells were determined by western blot analysis on days (A) 1, (B) 3 and (C) 13 of CCI. Protein blots were measured by densitometry. (D) The density of each p-p38 MAPK blot was normalized against that of total p38 MAPK to obtain a relative p-p38 MAPK blot density. Lane 1, sham/saline; lane 2, sham/etanercept; lane 3, CCI/saline; lane 4, CCI/etanercept. ^#^P<0.01 vs. the sham/saline group; ^*^P<0.01 vs. the CCI/saline group (n=4).

Western blot analysis showed that the HMGB1 protein expression (Fig. 3) and phosphorylated p38 MAPK levels (Fig. 4) were significantly induced by CCI on days 7 and 13 after surgery compared with those of the sham/saline and sham/etanercept groups,. Although etanercept showed no significant effect on the sham group, it significantly decreased the HMGB1 protein expression (Fig. 3) and phosphorylated p38 MAPK levels induced by CCI (Fig. 4).

## Discussion

Etanercept, a TNF-α inhibitor, reportedly exerts therapeutic effects on neuropathic pain in a rat CCI model (4). In the present study, we report for the first time that etanercept significantly decreased HMGB1 expression in DRG neuron cells in a rat CCI model, which provides fresh insights into the molecular mechanism underlying the therapeutic effect of etanercept on sciatica-related nociception.

HMGB1, which is abundantly expressed and presented in virtually all human cell types (13), functions as a cytokine (5). The induction of HMGB1 in DRGs reportedly contributes to pain hypersensitivity following peripheral nerve injury (8). In line with these reports, our study demonstrated that CCI significantly induced HMGB1 expression in DRG neuron cells, as well as thermal hyperalgesia and mechanical hyperalgesia.

TNF-α plays a pivotal role in CCI and sciatica-related nociception (1) and is upregulated in DRG neurons following spinal cord injury (2). It has been reported that HMGB1 is released from activated macrophages partly in a TNF-dependent manner (14). Our study demonstrated that etanercept, a TNF-α inhibitor, significantly decreased HMGB1 expression in DRG neuron cells, providing indirect evidence that TNF-α regulates HMGB1 expression in DRG neurons.

p38 MAPK is activated by phosphorylation at Thr180 and Tyr182 in response to inflammatory cytokines and stress, making it an important enzyme in diseases such as asthma and autoimmune disorders, as well as in the stress response of the nervous system (15). Kawahara *et al*(16) revealed that p38 MAPK is required for the active release of HMGB1 in macrophage cells. Kikuchi *et al*(17) reported that HMGB1 release from PC12 neuronal cells was blocked by a p38 MAPK inhibitor. In the current study, etanercept significantly decreased the phosphorylated p38 MAPK (Thr180 and Tyr182) level and HMGB1 expression induced by CCI, suggesting that etanercept inhibited HMGB1 expression by suppressing the activation of p38 MAPK. As TNF-α reportedly induces activation of p38 MAPK in DRG neurons (18), our findings provide indirect evidence that TNF-α regulates HMGB1 expression in DRG neurons through the p38 MAPK signaling pathway.

In conclusion, etanercept significantly reduced the HMGB1 expression induced by CCI in DRG neuron cells. This study not only explored the molecular mechanism underlying the therapeutic effect of etanercept on sciatica-related nociception, but also provided indirect evidence for the interaction between TNF-α and HMGB1 in DRG neuron cells.
